# A systematic review and meta—analysis of the prevalence and associated factors of iron—deficiency anemia among Chinese children under 6 years of age

**DOI:** 10.3389/fped.2025.1674121

**Published:** 2025-10-27

**Authors:** Weiwei Li, Yao Gong, Yan Lai, Yu Mao, Enlian Wu, Guolian Feng, Chun Yang, Jia Wang

**Affiliations:** Department of Child Healthcare, Pidu District People’s Hospital, Chengdu, China

**Keywords:** childhood, risk factors, prevalence, meta-analysis, China, iron-deficiency anemia

## Abstract

**Background:**

This meta-analysis aimed to determine the prevalence of iron-deficiency anemia (IDA) in Chinese children under 6 years of age and identify the associated risk factors.

**Methods:**

A comprehensive search was conducted for studies published up to 15 January 2025 in English and Chinese databases (PubMed, Embase, Web of Science, CNKI, and WanFang Data). A random-effects model was used to synthesize prevalence data and risk factors.

**Results:**

Twenty-three studies involving 93,570 children (19,994 with IDA) were included. The overall IDA prevalence was 20.61% (95% CI: 14.68%–26.54%), with higher rates in rural areas (29.96%) compared to urban areas (13.28%). Significant risk factors for IDA included male sex (OR = 1.35), age 6-12 months (OR = 4.10), age 13–24 months (OR = 2.66), low birth weight (<2,500 g) (OR = 1.46), maternal anemia during pregnancy (OR = 2.50), cesarean delivery (OR = 1.18), and premature birth (OR = 2.15). Protective factors included mixed feeding (OR = 0.59), artificial feeding (OR = 0.54), and early introduction of complementary feeding (<6 months) (OR = 0.57). Respiratory diseases, diarrhoea, and formula feeding were not significant risk factors. Meta-regression showed no impact of study design or sample size on these associations.

**Conclusion:**

Risk factors for IDA in Chinese children under 6 years of age include male sex, younger age (6–24 months), low birth weight, maternal anemia, cesarean delivery, and premature birth. Mixed or artificial feeding and early complementary feeding may protect against IDA. Further large-scale worldwide studies are needed to confirm these findings and inform public health strategies.

## Introduction

Iron-deficiency anemia (IDA) is the most common nutritional deficiency worldwide, disproportionately affecting children in low- and middle-income countries. According to the World Health Organization, approximately 42% of children under 5 years globally are anemic. In China, the prevalence of all-cause anemia has been reported to range from 20% to 53%, exceeding the global average in many regions (1, 2). The high burden of anemia in young children in China can be attributed to several socio-economic factors, including poverty, insufficient dietary iron intake, and disparities in healthcare access. Despite efforts to mitigate anemia through national health programs, such as iron supplementation and fortification, the prevalence remains disturbingly high, necessitating further investigation into the underlying causes and effective interventions (2).

IDA exerts a substantial negative impact on child health and development, particularly during the first years of life when rapid growth increases physiological iron requirements (3, 4). Iron deficiency in childhood is typically caused by inadequate dietary iron intake, rapid growth, and increased susceptibility to infections, which deplete iron stores. When iron deficiency progresses, hemoglobin synthesis is impaired, ultimately resulting in anemia. Children with IDA may suffer impaired cognitive development, delayed motor skills, and increased susceptibility to infections (5, 6). Furthermore, IDA has been linked to increased morbidity and mortality in children, particularly in regions where access to healthcare is limited (7).

The causes of anemia in young children are multifactorial. Beyond nutritional inadequacies, maternal anemia during pregnancy, low birth weight, and preterm birth contribute to reduced neonatal iron stores (8, 9). Infant feeding practices, including exclusive breastfeeding beyond 6 months without appropriate complementary foods, may further exacerbate iron deficiency (10, 11). Additionally, children living in rural areas or from socio-economically disadvantaged households are disproportionately affected due to limited access to iron-rich foods and healthcare services (1).

Although many of these determinants are globally recognized, some inconsistencies remain in the Chinese literature. These discrepancies often reflect geographic variation, dietary patterns, and socio-economic differences rather than a lack of consensus on the major risk factors. For example, studies from rural western provinces have reported higher anemia prevalence and stronger associations with nutritional inadequacies, while reports from urban eastern regions have emphasized maternal anemia and cesarean delivery as important contributors (1). Therefore, while a consensus exists on many determinants of childhood IDA, the regional and contextual variations in China necessitate a systematic synthesis of the evidence. This systematic review and meta-analysis aim to synthesize the existing evidence on the prevalence of IDA and its associated risk factors in Chinese children under 6 years of age.

## Material and methods

### Study design and database search

This systematic review and meta-analysis were conducted following the Preferred Reporting Items for Systematic Reviews and Meta-Analyses (PRISMA) guidelines (12). A comprehensive search strategy was developed to identify all relevant studies published up to 15 January 2025 on the prevalence of IDA in children under 6 years of age in China. The search was performed in multiple electronic databases, including PubMed, Embase, Web of Science, China National Knowledge Infrastructure (CNKI), and WanFang Data. Keywords used in the search included “iron-deficiency anaemia OR iron-deficiency anemia,” “children OR child OR infant OR pediatric OR paediatric OR preschool,” “prevalence,” “risk factors,” and “China.” Studies published in both English and Chinese languages were considered, and no restrictions were applied to the publication date or study design. All retrieved studies were imported into EndNote X9 (Thomson Reuters, New York, USA) for reference management and duplicates were removed. Detailed literature search strategies are provided in Supporting Information Supplementary Appendix 1.

### Inclusion and exclusion criteria

Studies were included in the meta-analysis if they met the following criteria: (1) original peer-reviewed research articles; (2) studies that reported the prevalence of IDA in children under 6 years of age; (3) studies conducted in mainland China; (4) studies that reported data on risk factors associated with IDA, such as demographic, nutritional, or health-related factors. Studies were excluded if they met any of the following criteria: (1) not peer-reviewed publications (e.g., conference abstracts, dissertations, or reports without full peer review); (2) studies focused on populations outside of mainland China; (3) studies that did not provide sufficient data on the prevalence of IDA or associated risk factors; (4) animal studies, reviews, or meta-analyses; (5) studies with incomplete or unreliable data. Two independent reviewers screened the titles, abstracts, and full texts of all identified articles to ensure the inclusion criteria were met.

### Data extraction

Data extraction was performed by two independent reviewers using a pre-designed data collection form. The following data were extracted from each included study: (1) basic study characteristics, including author(s), year of publication, region of study, and study design; (2) sample size; (3) prevalence of IDA in the study population, stratified by age group, sex, and other relevant subgroups; (4) information on associated risk factors for IDA, such as low birth weight, maternal anemia, premature birth, feeding practices, and socio-economic factors. Discrepancies between the two reviewers in the data extraction process were resolved through discussion, and if necessary, by consulting a third reviewer. Data were cross-checked with the original publications to ensure accuracy and consistency.

### Quality assessment

The methodological quality of the included studies was assessed using the Newcastle-Ottawa Scale (NOS) for observational studies (13). The NOS evaluates studies based on three broad categories: selection of study participants, comparability of study groups, and assessment of outcome. Each study was assigned a score based on the quality of its methodology, with a higher score indicating better methodological rigor. Studies that scored 7 or more points were considered high quality, those scoring 4–6 points were categorized as moderate quality, and studies with scores of 3 or fewer were considered low quality (13). Two independent reviewers performed the quality assessment, and disagreements were resolved by discussion or consultation with a third reviewer.

In addition to the Newcastle-Ottawa Scale, we assessed the overall certainty of evidence for each outcome using the Grading of Recommendations, Assessment, Development and Evaluation (GRADE) approach (14). The GRADE framework evaluates five domains: risk of bias, inconsistency, indirectness, imprecision, and publication bias. Evidence was rated as high, moderate, low, or very low certainty. Two reviewers independently performed the GRADE assessment, and disagreements were resolved through discussion or by consultation with a third reviewer. A summary of findings and evidence profile was generated using GRADEpro GDT software (McMaster University, 2020) and is presented in Supplementary Appendix 11.

### Statistical analysis

Statistical analyses were performed using the statistical software STATA 12.0 (StataCorp, College Station, TX, USA). The pooled prevalence of IDA and its risk factors in children under 6 years of age was calculated using a random-effects model, which accounts for China's vast population, regional variations in economic development, and differences in age groups across the studies. The *I*^2^ statistic was used to assess the degree of heterogeneity, with values greater than 50% indicating substantial heterogeneity (15). In the absence of frequency data, odds ratio (OR), risk ratio (RR) with 95% confidence intervals (CIs) were collected. RRs were converted to ORs using the formulas: OR=RR×(1−p0)/(1−RR×p0), where *P_0_* is the incidence in the non-exposed group (16). Sensitivity analyses were performed to assess the robustness of the results by excluding each study at a time. Publication bias was assessed with the Begg's (17) and Egger's (18) test. A two-tailed *P*-value of less than 0.05 was considered statistically significant.

### Meta-regression analyses

Meta-regression was performed to explore the impact of study-level variables on the observed heterogeneity in the prevalence estimates. Variables considered in the meta-regression analysis included study design (e.g., cross-sectional, case-control study, cohort) and sample size (<1,000, 1,000–5,000, ≥5,000). In the regression model, outcome served as dependent variable (y) while covariates acted as independent variables (*χ*). The meta-regression model was run using the “metareg” command in STATA.

## Results

### Study selection

**Figure 1:**
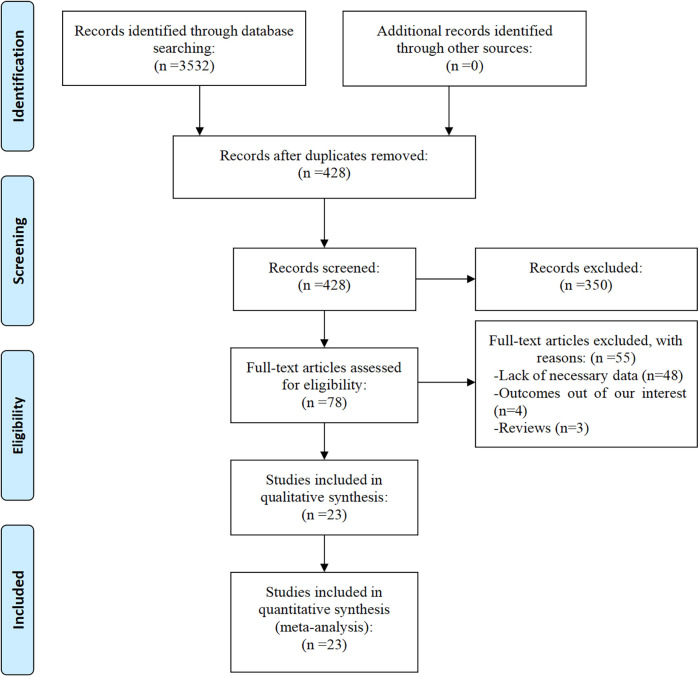
Eligibility of studies for inclusion in meta-analysis.

A total of 3,532 articles were initially identified from the databases PubMed, Embase, Web of Science, CNKI, and WanFang Data. After the removal of duplicates, 428 articles remained. Following a detailed screening of titles and abstracts, 78 full-text articles were assessed for eligibility. Of these, 55 were excluded for the following reasons: lack of necessary data (*n* = 48), absence of relevant outcomes (*n* = 4), and article type being reviews (*n* = 3). Finally, 23 studies (19–41) met the inclusion criteria and were ultimately included in the meta-analysis. Figure 1 presents the flow diagram of the study selection process.

### Characteristics of studies and quality assessment

The 23 studies included in the meta-analysis consisted of 93,570 children, 19,994 of whom were diagnosed with IDA (Table 1). The studies were conducted across various regions of China, with publication years ranging from 2011 to 2024. Study designs were diverse, including cross-sectional (*n* = 15), case-control studies (*n* = 5) and cohort (*n* = 3). The sample size of the studies ranged from 94 to 24,235 children. The studies were conducted in various geographic regions of China, including urban and rural settings, reflecting the diversity in socio-economic conditions and healthcare access. Among the 8 cohort and case-control studies, 3 were classified as high quality (NOS score ≥7) (21, 23, 24), 4 as moderate quality (NOS score 4–6) (31, 33, 38, 41), and 1 as low quality (NOS score <4) (32) (Supplementary Appendix 11). The majority of studies had robust methodologies, though some exhibited potential biases, such as small sample sizes or lack of detailed reporting on confounding factors.

**Table 1:** Baseline characteristics of patients in the trials included in the meta-analysis.

Study	Design	Sample size	Setting	Age (month)	Prevalence of anaemia (%)	NOS score
Wang J (19)	Case-control	294	An Urban Community in Shanghai	0–24	11.49	7
Yang W (20)	Cross-sectional	336	Rural areas of Shaanxi	0–18	35.12	NA
Clark KM (21)	Cohort	955	Zhejiang AND Hebei	9	NR	7
Huang Y (22)	Cross-sectional	754	Rural areas of Qinghai	6–23	59.1	NA
Xin QQ (23)	Cohort	24,235	11 province-level regions	0–36	24.4	8
Wang J (24)	Cross-sectional	1,370	Poor rural areas of China	0–36	25.6	NA
Li H (25)	Cross-sectional	5,229	Rural Hunan Province	0–71	8.8	NA
Zhou XT (26)	Cross-sectional	93	Hospital outpatient in Beijing	6–36	26.0	NA
Li H (27)	Cross-sectional	420	Urban areas of Shanghai	0–6	12.6	NA
Chen M (28)	Cross-sectional	190	Rural areas of Shanghai	0–6	21.58	NA
Liu Y (29)	Cross-sectional	1,026	Urban areas of Shanghai	0–24	14.62	NA
Du YJ (30)	Cross-sectional	7,899	Urban areas of Jinan	6–72	5.28	NA
Yu JJ (31)	Cohort	449	Urban areas of Foshan	0–36	18.49	6
Yu JY (32)	Case-control	236	Dongguan areas	36–72	NR	3
Hou SY (33)	Case-control	182	Rural areas of Shanghai	0–72	23.6	5
Meng HX (34)	Cross-sectional	200	Rural and Urban areas of Beijing	6–24	18.5	NA
Yu CY (35)	Cross-sectional	19,498	Rural areas of Qiannan in Guizhou	6–23	47.59	NA
Li HW (36)	Cross-sectional	130	Rural areas of Yunnan	6–9	33.8	NA
Zhang YX (37)	Cross-sectional	220	Urban areas of Ma’anshan	0–36	7.5	NA
Jin H (38)	Case-control	1,477	Urban areas of Suzhou	6	18.3	6
Li ZF (39)	Cross-sectional	2,215	Urban areas of Shangluo	0–72	19.68	NA
Wang ZC (40)	Cross-sectional	3,503	Urban areas of Suzhou	36–48	7.5	NA
Zhang J (41)	Case-control	22,659	Urban areas of Tianjin	6–8	6.69	6

NOS, Newcastle-Ottawa Scale; NR, not reported; NA, not available.

Furthermore, a GRADE assessment was performed to evaluate the certainty of evidence. Overall, the certainty was rated as low to very low, primarily due to risk of bias and inconsistency. As all included studies were observational in design, the quality of evidence remained low despite some outcomes demonstrating a large magnitude of effect (pooled OR >2). Detailed evidence profiles are provided in Supplementary Appendix 12.

### Prevalence of IDA

**Figure 2:**
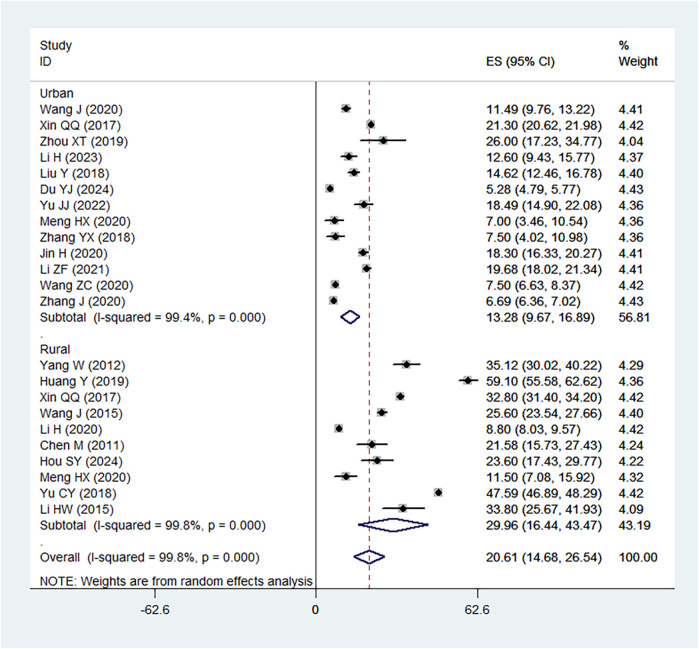
Forest plot showing the prevalence of IDA in children under 6 years of age in China.

The pooled prevalence of IDA in children under 6 years of age in China was estimated to be 20.61% (95%CI: 14.68%–26.54%), with considerable heterogeneity observed between studies (*I*^2^ = 99.8%) (Figure 2). The prevalence ranged from a low of 5.28% to a high of 59.1%, reflecting regional differences and variations in study design, population characteristics, and diagnostic criteria. Subgroup analysis showed that prevalence in in rural areas was 29.96% (95%CI: 16.44%–43.47%), which was higher than that in urban areas (13.28%, 95%CI: 13.28%–16.89%).

Several individual studies reported substantially higher or lower prevalence compared with the pooled estimate. For instance, Huang Y (highest prevalence rate, 59.10%), Yu CY (47.59%) and Yang W (35.12%) observed high prevalence rates, which may be attributable to sampling from rural western provinces where nutritional deficiencies are more severe and healthcare access is limited. In contrast, Du YJ reported a relatively low prevalence (<10%), likely reflecting an urban population with higher socioeconomic status and better maternal-child health services. These findings highlight how geographic and socioeconomic contribute to the variability in prevalence across studies.

### Risk and protective factors for IDA

**Figure 3:**
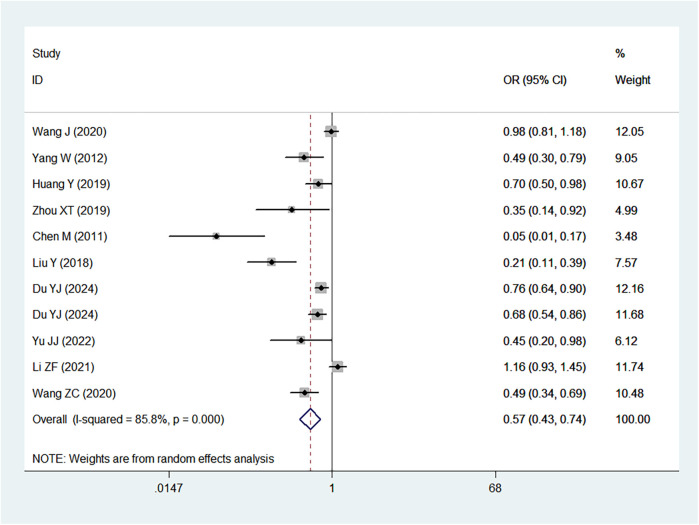
Forest plot showing the relationship between premature birth and IDA.

**Figure 4:**
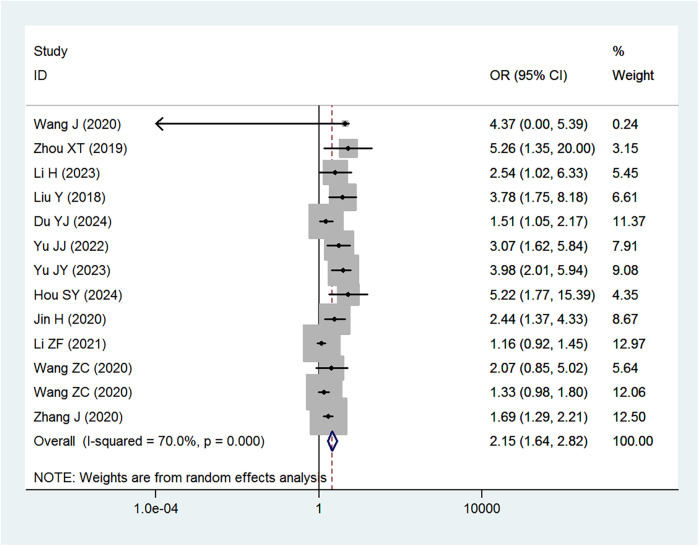
Forest plot showing the relationship between maternal anemia during pregnancy and IDA.

Table 2 provides a summary of the risk and protective factors for IDA. Premature birth (Figure 3), maternal anemia during pregnancy (Figure 4), low birth weight (Supplementary Appendix 2), male sex (Supplementary Appendix 3), cesarean delivery (Supplementary Appendix 4), and younger age (6–24 months) (Supplementary Appendix 5) were significantly associated with higher risk of IDA.

**Table 2:** Summary of pooled associations between risk factors and IDA in children under 6 years of age in China.

Risk factor	No. of studies	Pooled OR (95% CI)
Premature birth	12	2.15 (1.64–2.82)
Maternal anemia in pregnancy	12	2.50 (1.62–3.85)
Low birth weight (<2,500 g)	10	1.46 (1.23–1.72)
Male sex	7	1.35 (1.17–1.56)
Age 6–12 months	6	4.10 (1.68–10.01)
Age 13–24 months	6	2.66 (1.10–6.47)
Mixed feeding	14	0.59 (0.43–0.79)
Artificial feeding	14	0.54 (0.36–0.83)
Early complementary feeding	10	0.57 (0.43–0.74)
Cesarean delivery	4	1.18 (1.00–1.39)
Diarrhea	4	1.29 (0.99–1.68)
Respiratory disease	4	1.30 (0.92–1.84)
Early initiation of breastfeeding	2	1.00 (0.70–1.43)

**Figure 5:**
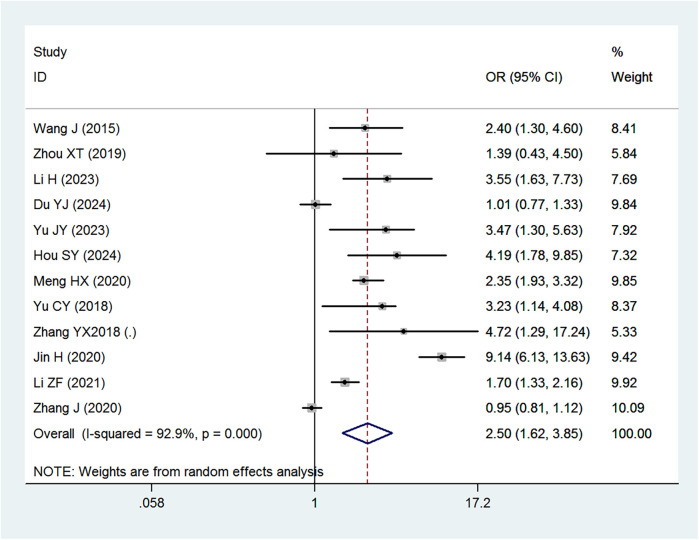
Forest plot showing the relationship between introduction of complementary feeding and IDA.

Mixed or artificial feeding (Supplementary Appendix 6), as well as early introduction of complementary foods (Figure 5), were associated with reduced risk of IDA compared with exclusive breastfeeding. Neither diarrhea nor respiratory disease (Supplementary Appendix 7) were significantly associated with IDA.

### Sensitivity analysis

Sensitivity analyses were conducted for premature birth, maternal anemia during pregnancy, and the introduction of complementary feeding (Supplementary Appendixes 8–10). The results indicated that removing any individual study did not alter the overall effect size. Specifically, for the outcome of preterm birth, the sensitivity analysis plot (Supplementary Appendix 8) showed that the lower 95% CI range of the total effect size, after removing any study, ranged from 1.96 to 2.14, while the upper range was between 3.33 and 3.58. Both the upper and lower 95% CIs exceeded 1, suggesting that the exclusion of any study did not impact the overall effect size. This demonstrates that, despite heterogeneity, the results remain robust.

### Meta-regression

Meta-regression analyses were conducted to examine the influence of study-level variables on the pooled associations between risk factors and IDA. Results indicated that sample size did not significantly affect the associations for mixed feeding vs. breastfeeding (*P* = 0.068), maternal anemia during pregnancy (*P* = 0.356), premature birth (*P* = 0.360), birth weight (*P* = 0.322), and introduction of complementary feeding (*P* = 0.515). Similarly, sample size did not significantly impact the associations for mixed feeding vs. breastfeeding (*P* = 0.106), maternal anemia during pregnancy (*P* = 0.148), premature birth (*P* = 0.260), birth weight (*P* = 0.982), and introduction of complementary feeding (*P* = 0.362). These results are summarized in Table 3.

**Table 3:** Meta-regression analysis for the impact of potential factors on the outcomes.

Potential factors	Study design			Sample size		
	*β* coefficient (95%CI)	*P* value	*R*^2^, %	*β* coefficient (95%CI)	*P* value	*R*^2^, %
Mixed feeding vs. Breastfeeding	−0.93 (−1.95, 0.09)	0.068	4.64	0.75 (−0.19, 1.69)	0.106	−5.82
Maternal anemia during pregnancy	0.40 (−0.53, 1.32)	0.356	−2.58	−0.37 (−0.90, 0.16)	0.148	16.28
Premature birth	0.07 (−0.09, 0.22)	0.360	28.85	−0.08 (−0.22, 0.06)	0.260	35.55
Birth weight	−0.32 (−1.03, 0.39)	0.322	4.13	0.01 (−0.61, 0.63)	0.982	−9.37
Introduction of complementary feeding	0.31 (−0.73, 1.34)	0.515	−16.91	0.33 (−0.45, 1.10)	0.362	−14.01

95%CI, 95% confidence interval.

### Publication bias

Begg and Egger tests across all included studies showed no evidence of publication bias (Begg: *Z* = 0.62, *P* = 0.533; Egger: *t* = −0.84, *P* = 0.422) (Supplementary Appendix 13).

## Discussion

In this systematic review and meta-analysis, we evaluated the prevalence and associated risk factors of IDA in children under six years of age in China. The findings indicate that the pooled prevalence of IDA in this population is 20.61%, with substantial regional variability ranging from 5.28% to 59.1%. This estimate is consistent with previously reported prevalence rates of all-cause anemia in the range of 20%–53%, suggesting that IDA remains a major public health concern in China, particularly in rural areas. Moreover, several key risk factors associated with IDA were identified, including premature birth, maternal anemia during pregnancy, low birth weight, male sex, and inadequate infant feeding practices such as delayed introduction of complementary foods. Identifying these risk factors is crucial for the development of targeted interventions aimed at reducing the burden of IDA in young children.

The analysis revealed that premature birth (<37 weeks gestation) was significantly associated with an increased risk of IDA (OR = 2.15). This finding aligns with previous studies indicating that preterm infants are at a higher risk of IDA due to limited iron stores at birth and increased iron requirements during rapid postnatal growth (42, 43). A study by Ruan et al. demonstrated that preterm infants have significantly lower iron reserves, leading to a higher incidence of IDA in early childhood (42). Similarly, Moreno-Fernandez et al. reported that premature infants are more likely to develop iron deficiency and anemia during their first year of life, largely due to reduced placental iron transfer and an earlier onset of growth spurts that rapidly deplete iron stores (43).

Maternal anemia during pregnancy was also identified as a strong risk factor for IDA in children (OR = 2.50), consistent with prior research demonstrating that maternal iron deficiency is a critical determinant of offspring IDA (10). A study by Abioye et al. found that maternal IDA was associated with a significantly increased risk of infant anemia at six months of age (10). This association is attributed to reduced iron transfer from the mother to the fetus, resulting in lower neonatal iron stores and an increased likelihood of developing IDA in infancy and early childhood (44).

Cesarean delivery was found to be modestly associated with a higher risk of IDA (OR = 1.18). Although the effect size was relatively small, this finding is biologically plausible. One explanation is that delayed cord clamping, which increases neonatal iron stores, is less frequently practiced during cesarean sections compared to vaginal deliveries. Additionally, cesarean delivery has been linked to altered establishment of the infant gut microbiota, which may influence nutrient absorption, including iron. Moreover, maternal complications leading to cesarean delivery, such as pregnancy-related anemia or preeclampsia, could indirectly affect neonatal iron status. These potential mechanisms warrant further prospective studies.

Low birth weight (<2,500 g) was significantly associated with an increased risk of IDA (OR = 1.46), consistent with findings from previous studies (45). Berglund et al. (45) observed that healthy marginally low birth weight infants, whether preterm or term, exhibited a high risk of developing iron deficiency (36%) and IDA (9.9%) at six months of age, particularly if they were exclusively breastfed (iron deficiency: 56%, IDA: 18%). The increased susceptibility to IDA among low birth weight infants can be attributed to their reduced iron stores at birth and higher iron demands during early postnatal growth. Additionally, a meta-analysis by Gedfie et al. found that children with low birth weight had a 1.15-fold higher risk of anemia compared to those with normal birth weight, supporting the present findings (46).

The results further indicate that both mixed feeding (OR = 0.59) and artificial feeding (OR = 0.54) were protective against IDA when compared to exclusive breastfeeding. These findings are consistent with prior research suggesting that mixed feeding provides a more balanced nutrient profile, including iron, compared to exclusive breastfeeding, which may not meet the increasing iron demands of growing infants. A study by Zhang et al. found that mixed feeding was associated with a lower risk of IDA in infants aged 6–8 months (41), while Xin et al. reported that infants exclusively breastfed for more than six months had a higher incidence of IDA compared to those who received early complementary foods (23). However, conflicting results have been reported in some studies. For instance, Meng et al. found no significant difference in IDA prevalence between exclusively breastfed infants and those receiving mixed feeding (OR = 1.021, 95% CI: 0.864–1.211) (34). Such discrepancies may be attributed to variations in the timing and quality of complementary food introduction, as well as differences in the iron content of these foods.

Age-related differences in IDA prevalence were also observed, with children aged 6–12 months and 13–24 months at a significantly higher risk compared to those aged 25–36 months. These findings are in agreement with previous research highlighting that the rapid growth phase between 6 and 24 months represents a critical period of increased iron demand, making infants particularly vulnerable to IDA if adequate iron intake is not ensured. A study by Xin et al. reported that children aged 6–12 months exhibited the highest incidence of IDA, largely due to increased iron requirements and insufficient complementary feeding practices (23).

In many countries, anemia prevalence is typically higher among females, particularly during adolescence and reproductive years, due to menstrual blood loss and higher iron demands (47). However, our analysis among children under 6 years revealed that boys were more likely to develop IDA than girls (OR = 1.35). This finding is consistent with several other studies in early childhood populations, which suggest that male infants and toddlers may be more vulnerable to iron deficiency (48, 49). Possible explanations include the faster growth velocity and greater lean body mass accretion in boys, leading to higher iron requirements during infancy. Additionally, some evidence suggests that boys may have lower iron stores at birth compared with girls, predisposing them to earlier depletion (50). Cultural or nutritional factors may also contribute. In certain Chinese contexts, prolonged exclusive breastfeeding without timely iron supplementation has been more frequently reported among male infants (21). Conversely, some studies have not found a clear sex difference, indicating that the relationship may vary depending on dietary patterns, socioeconomic conditions, and study design. At present, the precise biological and environmental explanations remain uncertain, and further research is needed to clarify why boys appear more susceptible to IDA in early childhood in China.

Several factors, including early initiation of breastfeeding, diarrhea, and respiratory disease, were not significantly associated with IDA in our analysis. This may be due to several reasons. Early initiation of breastfeeding is crucial for neonatal immunity and bonding but breast milk alone contains relatively low iron content, and therefore may not directly prevent IDA without timely introduction of complementary foods. Similarly, diarrhea and respiratory diseases can influence short-term nutrient absorption and increase iron losses, but the available cross-sectional studies may not have captured these transient effects or may have lacked sufficient follow-up duration to detect long-term associations. As such, while biologically plausible, these nonsignificant associations may reflect study design limitations rather than a true absence of effect.

The findings of this study have important implications for clinical practice and public health policies in China. The high prevalence of IDA, particularly in rural areas, calls for increased awareness and intervention at both the individual and community levels. Clinicians should prioritize screening for IDA in high-risk groups, including children with low birth weight, premature birth, and those born to mothers with anemia during pregnancy. Iron supplementation, particularly for children in these high-risk categories, should be emphasized as part of routine care.

Furthermore, the results highlight the importance of early and appropriate introduction of complementary feeding. Pediatricians and healthcare providers should educate parents about the timing and types of complementary foods that are necessary to meet the increased iron requirements of infants after 6 months of age. Ensuring that infants receive iron-rich complementary foods can help reduce the incidence of IDA and improve overall child health outcomes.

While our study provides valuable insights into the prevalence and risk factors of IDA in Chinese children, further research is needed to deepen our understanding of the underlying causes of IDA. Future studies should focus on longitudinal designs to better assess the causal relationships between risk factors and IDA. Additionally, research on the effectiveness of different intervention strategies, such as iron supplementation programs and dietary interventions, is needed to identify the most effective ways to prevent and treat IDA in young children.

In particular, there is a need for more research on the role of breastfeeding practices in preventing IDA. While exclusive breastfeeding has been shown to offer numerous health benefits, its adequacy in preventing IDA remains uncertain in certain populations. Future studies should explore the impact of different breastfeeding patterns, including the duration and timing of introduction to complementary feeding, on the risk of IDA.

Despite its strengths, this meta-analysis has several potential limitations. First, the included studies exhibited significant heterogeneity, which may have influenced the pooled estimates. Variations in study design, sample size, geographic region, and diagnostic criteria for IDA could account for some of this heterogeneity. Although we conducted sensitivity analyses to assess the robustness of our findings, residual confounding may still exist. Second, some of the studies included in the analysis were of moderate or low methodological quality, which could introduce bias. For example, several studies lacked detailed reporting on confounding factors or had small sample sizes, which may affect the generalizability of the results. Lastly, the cross-sectional nature of many included studies limits our ability to establish causality between the identified risk factors and IDA.

In conclusion, this meta-analysis provides a comprehensive assessment of the prevalence of IDA and its associated risk factors in children under 6 years of age in China. The findings highlight the high burden of IDA, especially in rural areas, and identify several key risk factors, including premature birth, maternal anemia, low birth weight, and feeding practices. These results underscore the need for targeted interventions, such as improved maternal health care, iron supplementation programs, and education on infant feeding practices, to reduce the prevalence of IDA in young children. Further high-quality, longitudinal studies are needed to confirm these findings and explore effective strategies for the prevention and management of IDA.

## Data Availability

The original contributions presented in the study are included in the article/Supplementary Material, further inquiries can be directed to the corresponding author.
