# Supplementary material for: A systematic review and meta—analysis of the prevalence and associated factors of iron—deficiency anemia among Chinese children under 6 years of age
Source: Front Pediatr. 2025 Oct 27;13:1674121. doi: 10.3389/fped.2025.1674121 (PMC12598026; doi:10.3389/fped.2025.1674121)
Supplement: Supplementary file 1 [file Datasheet1.docx]

**Appendix S1. Search strategy**

Pubmed

1. "anemia, iron deficiency"[MeSH Terms] 12432
2. "anemia iron deficiency"[Title/Abstract] OR "anemias iron deficiency"[Title/Abstract] OR "anemias iron deficiency"[Title/Abstract] OR "iron deficiency anemias"[Title/Abstract] OR "iron deficiency anemias"[Title/Abstract] OR "iron deficiency anemia"[Title/Abstract] OR "iron deficiency anemia"[Title/Abstract] 9954
3. 1 OR 2 17811
4. "risk factors"[MeSH Terms] 1021196
5. "factor risk"[Title/Abstract] OR "risk factor"[Title/Abstract] OR "population at risk"[Title/Abstract] OR "populations at risk"[Title/Abstract] OR "risk scores"[Title/Abstract] OR "risk score"[Title/Abstract] OR "score risk"[Title/Abstract] OR "risk factor scores"[Title/Abstract] OR "risk factor score"[Title/Abstract] OR "score risk factor"[Title/Abstract] OR "health correlates"[Title/Abstract] OR "correlates health"[Title/Abstract] OR "social risk factors"[Title/Abstract] OR (("Factor"[All Fields] OR "factor s"[All Fields] OR "Factors"[All Fields]) AND "social risk"[Title/Abstract]) OR "factors social risk"[Title/Abstract] OR "risk factor social"[Title/Abstract] OR "risk factors social"[Title/Abstract] OR "social risk factor"[Title/Abstract] 351514
6. 4 OR 5 1230239
7. 3 AND 6 385
8. "china"[MeSH Terms] OR "china"[All Fields] OR ("mainland"[All Fields] AND "china"[All Fields]) OR "mainland china"[All Fields] 3337687
9. 7 AND 8 172

**Web of Science**

1. **TS=(**anemia iron deficiency OR anemias iron deficiency OR anemias iron deficiency OR iron deficiency anemias OR iron deficiency anemias OR iron deficiency anemia OR iron deficiency anemia**)**
2. **TS=(**factor risk OR risk factor OR population at risk OR populations at risk OR risk scores OR risk score OR score risk OR risk factor scores OR risk factor score OR score risk factor OR health correlates OR correlates health OR social risk factors)
3. **#1 AND #2**
4. **TS=(**china OR mainland china)
5. **#3 AND #4**

**EMBASE**

#1. 'anemia, iron deficiency'/exp

#2. 'anemia, iron deficiency':ab,ti

#3. 'Anemias, Iron-Deficiency':ab,ti

#4. ' Iron-Deficiency Anemias':ab,ti

#5. ' Iron Deficiency Anemia':ab,ti

#6. #1 OR #2 OR #3 OR #4 OR #5

#7. 'Factor, Risk':ab,ti

#8. 'Population at Risk':ab,ti

#9. 'Risk Scores':ab,ti

#10. 'Score, Risk':ab,ti

#11. 'Risk Factor Scores':ab,ti

#12. 'Score, Risk Factor':ab,ti

#13. 'Health Correlates':ab,ti

#14. 'Social Risk Factors':ab,ti

#15. 'Factors, Social Risk':ab,ti

#16. 'Risk Factor, Social':ab,ti

#17. 'Risk Factors, Social':ab,ti

#18. 'Social Risk Factor':ab,ti

#19. 'Risk Factor':ab,ti

#20. #7 OR #8 OR #9 OR #10 OR #11 OR #12 OR #13 OR #14 OR #15 OR #16 OR #17 OR #18 OR #19

#21. 'China':ab,ti

#22. 'Mainland China':ab,ti

#23. #21 OR #22

#24. #6 AND #20 AND #23

**
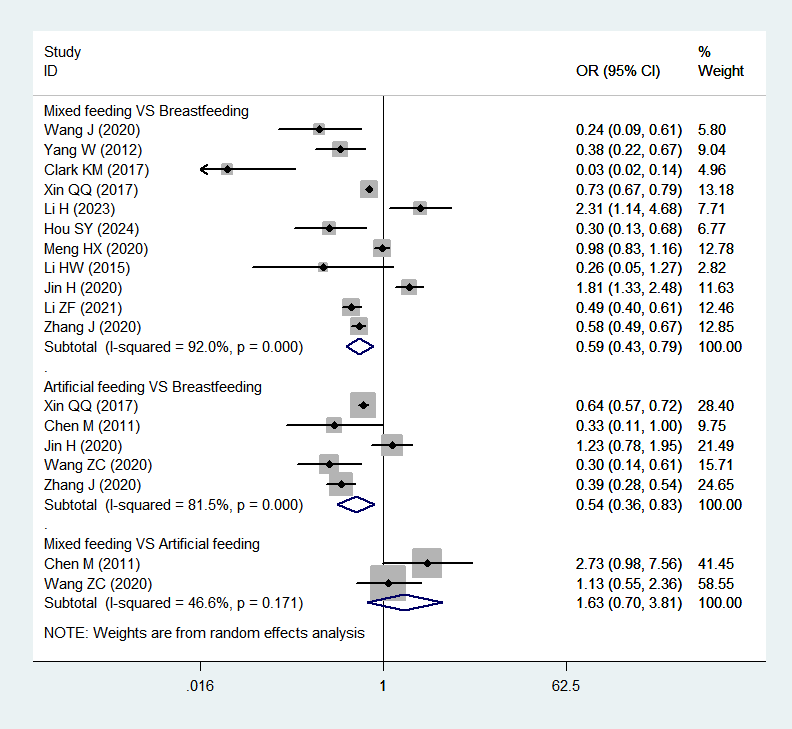
**

**Appendix S2. The relationship between Feeding patterns with IDA.**

**
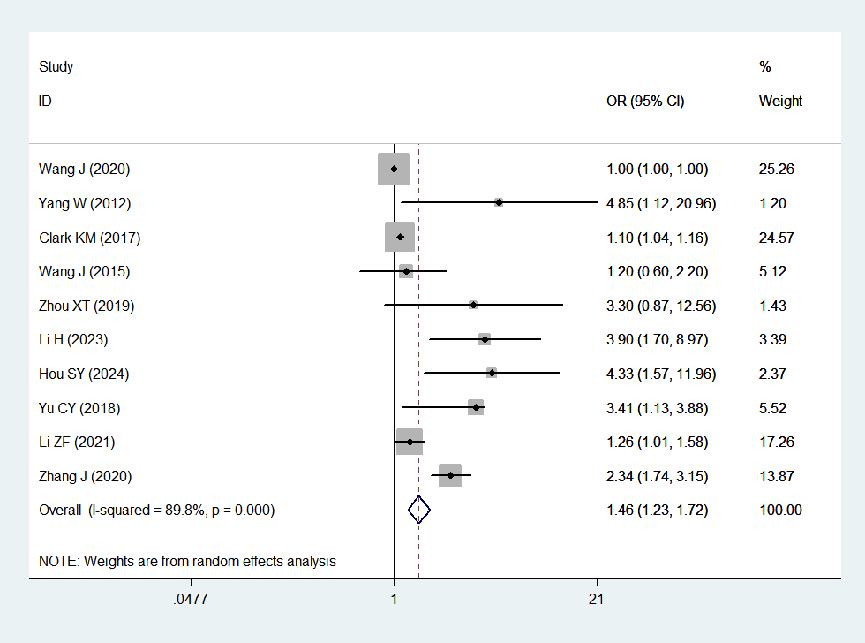
**

**Appendix S3. The relationship between birth weight with IDA.**

**
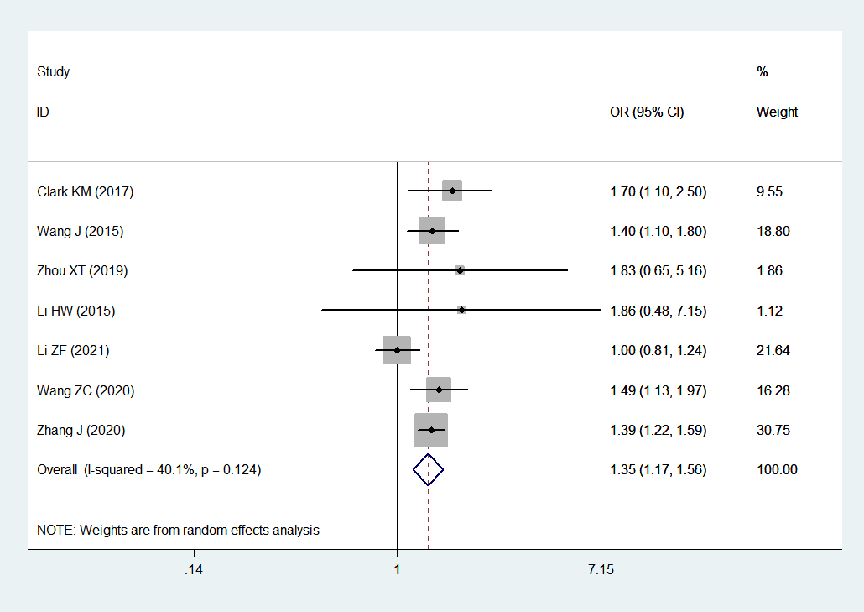
**

**Appendix S4. The relationship between male sex with IDA.**

**
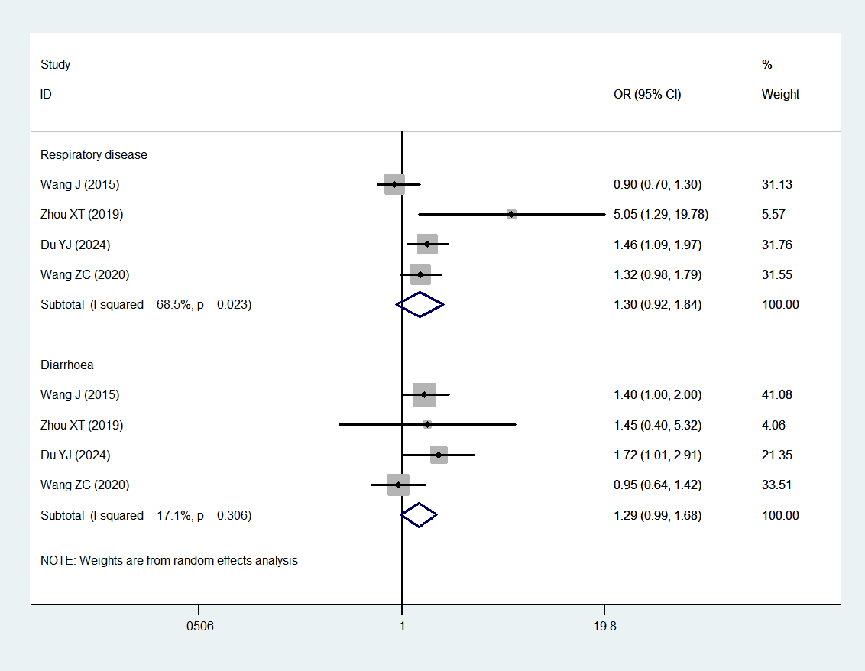
**

**Appendix S5. The relationship between diarrhoea/respiratory diseases with IDA.**

**
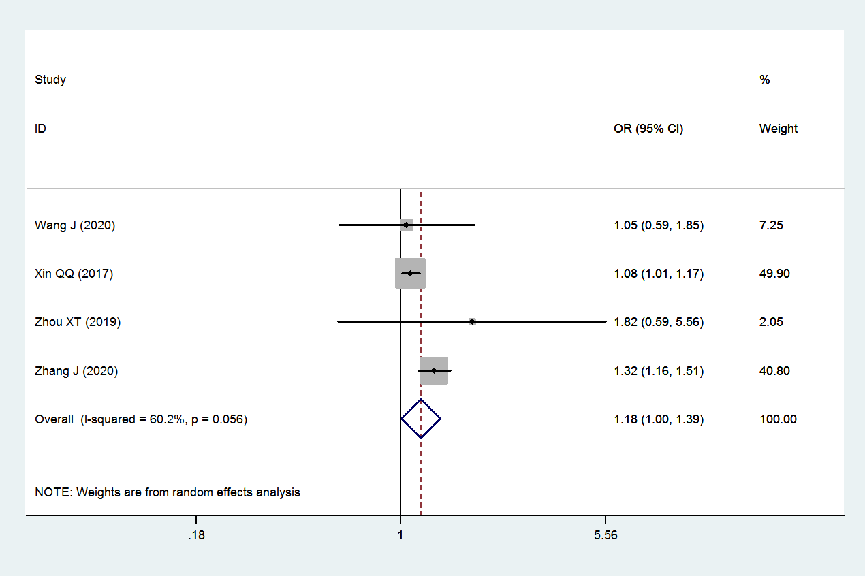
**

**Appendix S6. The relationship between mode of delivery with IDA.**

**
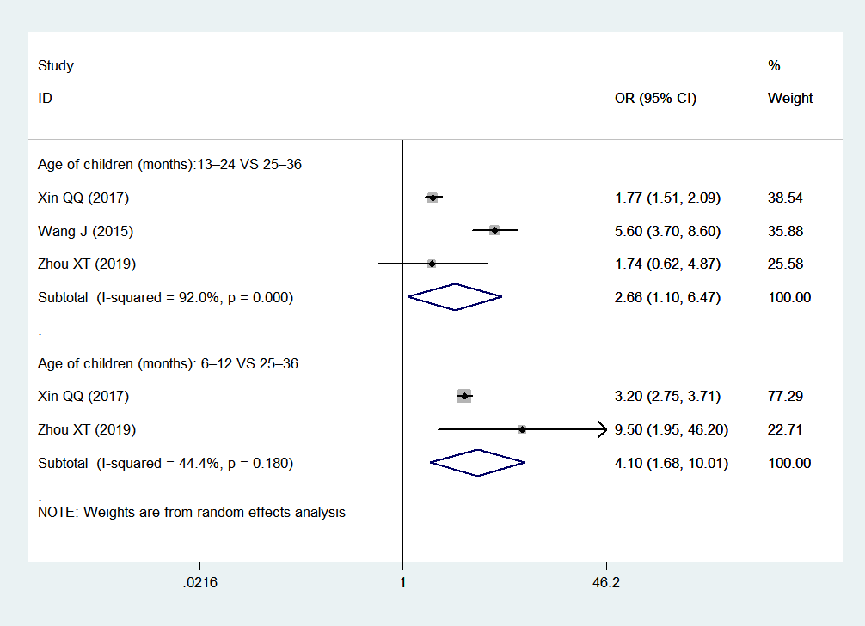
**

**Appendix S7. The relationship between age of children with IDA.**

**
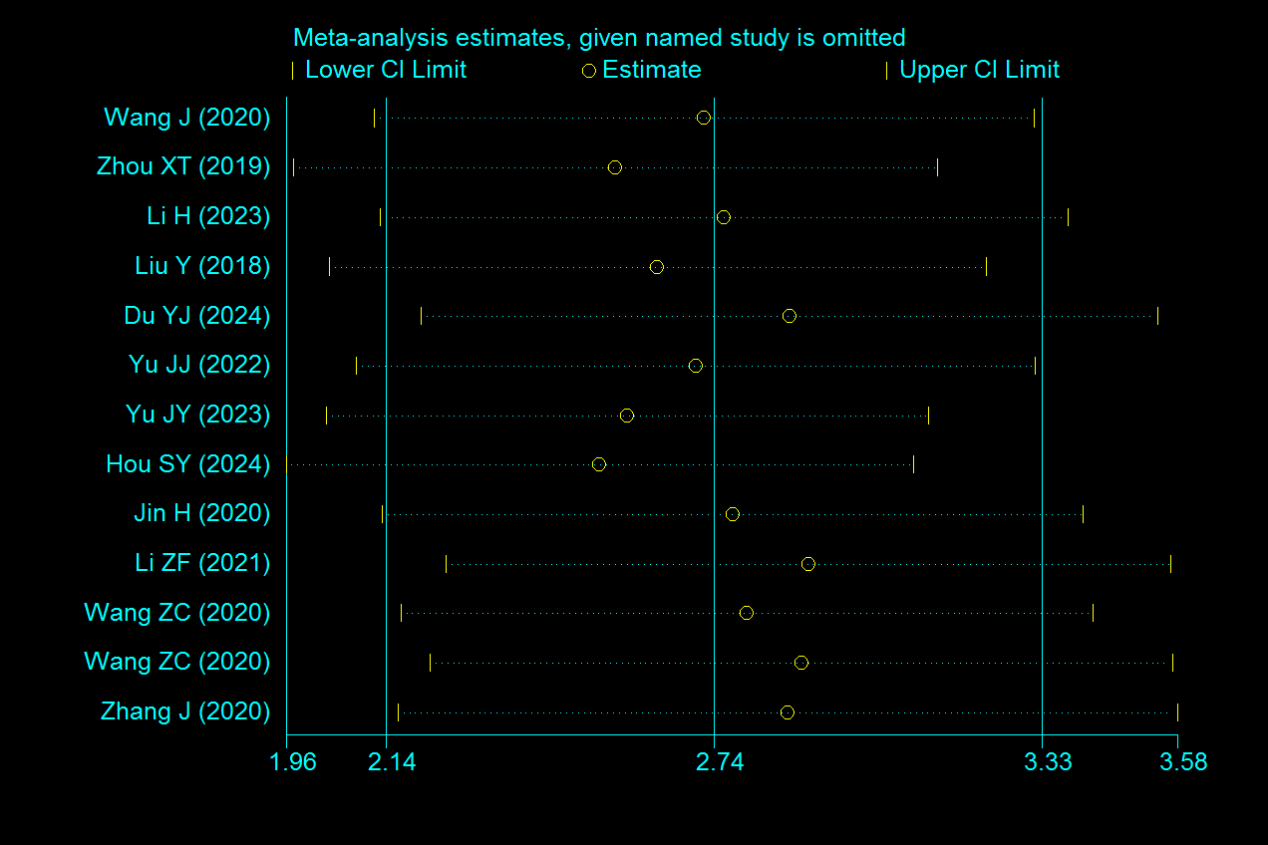
**

**Appendix S8. Sensitivity analysis for the relationship between premature birth with IDA.**

**
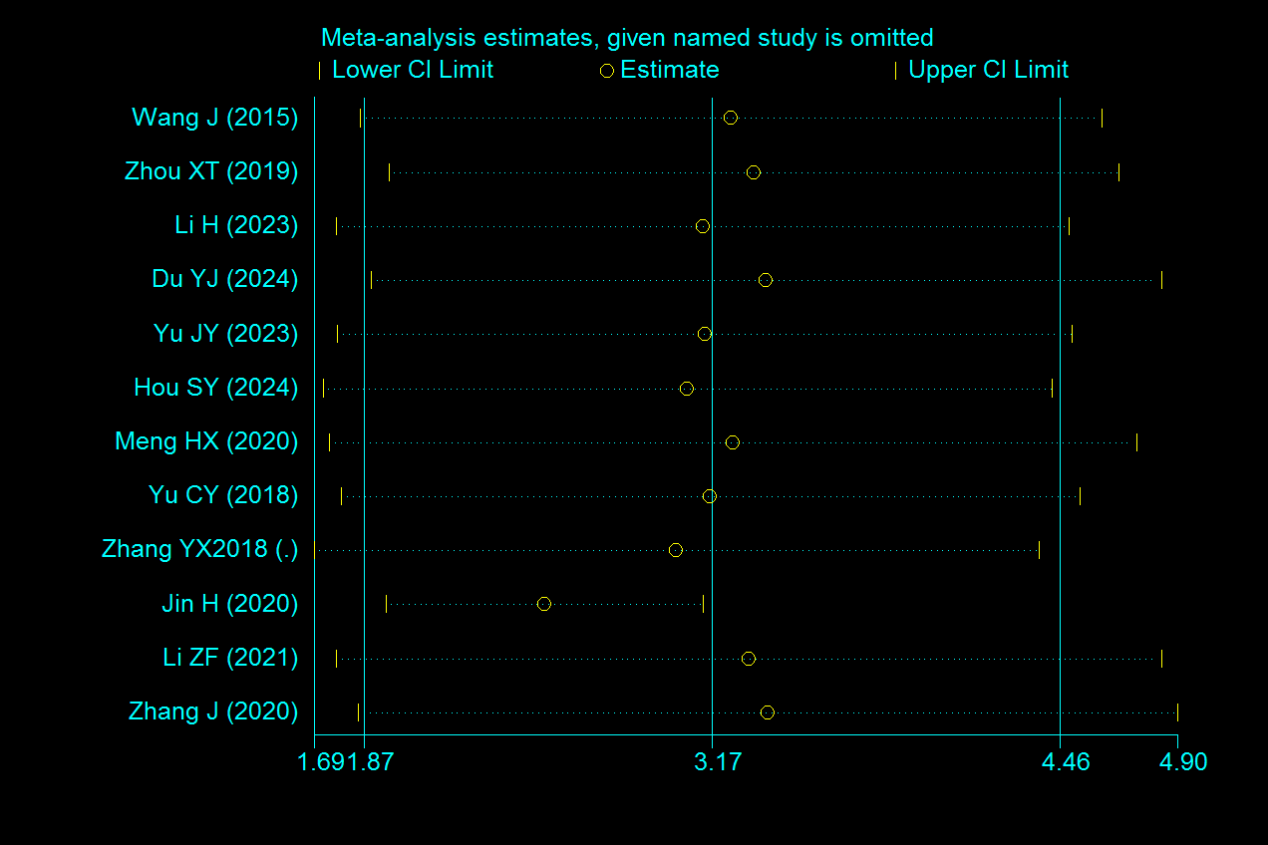
**

**Appendix S9. Sensitivity analysis for the relationship between maternal anemia during pregnancy with IDA.**

**
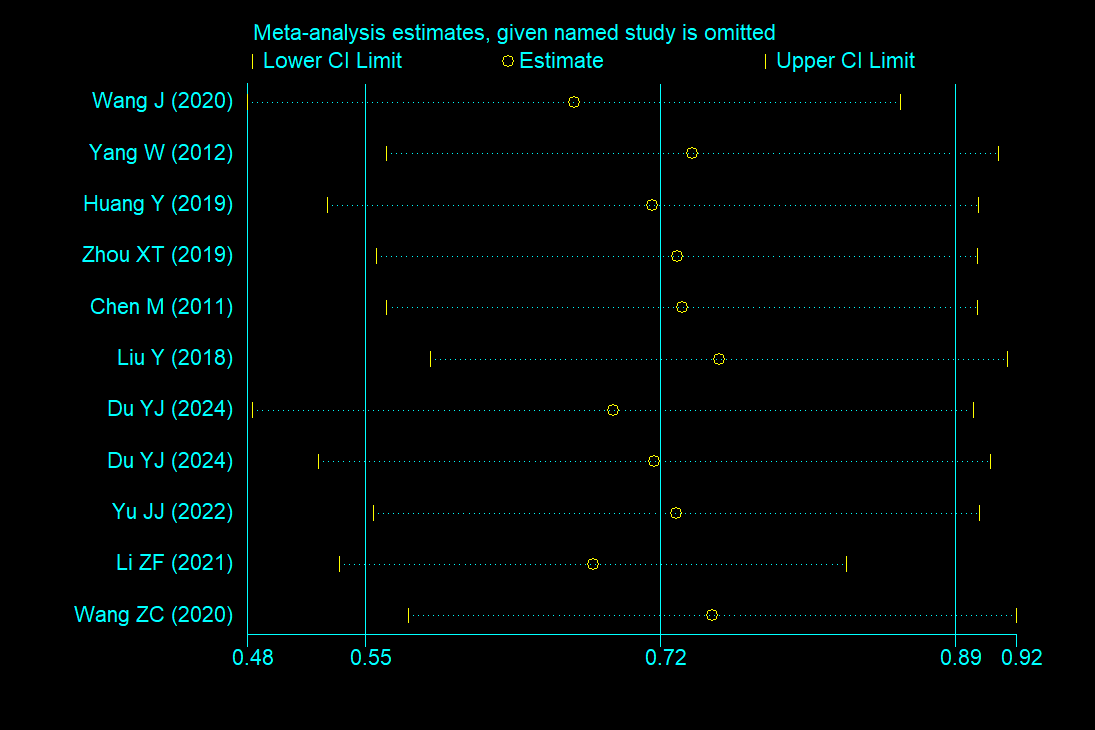
**

**Appendix S10. Sensitivity analysis for the relationship between introduction of complementary feeding with IDA.**
